# Self-esteem modulates the ERP processing of emotional intensity in happy and angry faces

**DOI:** 10.1371/journal.pone.0217844

**Published:** 2019-06-06

**Authors:** Jianfeng Wang, Yan Wu

**Affiliations:** 1 Department of Psychology, Chengdu Medical College, Chengdu, China; 2 Sichuan Research Center for Applied Psychology, Chengdu Medical College, Chengdu, China; Sapienza University of Rome, ITALY

## Abstract

Previous studies have shown that self-esteem modulates attentional responses to emotional stimuli. However, it is well known that emotional stimuli can vary in intensity. The main objective of the present study was to further investigate self-esteem related emotional intensity processing in happy and anger faces. Event-related potentials (ERPs) were recorded while 27 high-esteem versus 27 low self-esteem participants carried out a visual oddball task, with neutral faces as the standard stimuli and deviant stimuli varying on valence (happy and anger) and intensity (40%, 70%, and 100% emotive) dimensions. The results showed only high self-esteem people, instead of those with low self-esteem, displayed significant emotion intensity effects for 100% than for 70% happy faces in P3 component. On the other hand, only people with low self-esteem exhibited pronounced intensity effects for anger faces in P3 amplitudes. Moreover, only people with low self-esteem displayed significant intensity effects for 100% compared to both 70% and 40% anger stimuli in N2 amplitudes at central sites. These findings indicate that high self-esteem individuals were typically more susceptible to highly as well as mildly positive stimuli yet less reactive to negative stimuli compared with people with low self-esteem.

## Introduction

Self-esteem is deemed as one’s evaluation of self-knowledge that signals to what extent people accept and like themselves. High self-esteem means greatly favorable evaluation of oneself on the whole, while low self-esteem is associated with mildly positive or ambivalent feelings toward oneself [1]. Numerous studies have indicated that the difference in the level of self-esteem can influence how individuals respond to certain types of emotional information, such as emotional information concerning acceptance or rejection. For example, individuals with low self-esteem are more likely than those with high self-esteem to anticipate rejection [2], devote more attentional resources to potential rejection cues [3–6], fail to engage in strategies to prevent rejection [7], and react more strongly when rejection actually occurs in terms of self-reported responses [8] and physiological reactions [9,10].

However, emotional information in daily life often vary in intensity. For example, anger expressions can very much vary in intensity ranging from mild anger to rage. In fact, the intensity of emotional stimuli is important, and emotions of diverse strengths have different effects on the cognitive processes [11–13]. Using an oddball paradigm, in which subjects are asked to make a standard/deviant distinction, previous studies have reported a valence intensity effect that human brain is sensitive to valence differences in emotional stimuli [11]. Consistent with these findings, using facial expressions as materials, studies that employed overt [12] or covert [13] emotional tasks jointly showed increased neural responses of the brain to negative facial expressions of higher intensity. Although the difference in the level of self-esteem can influence how individuals respond to emotional information, whether response sensitivity increases with the intensity of emotional stimuli, and how individuals with high and low self-esteem differ in processing valence intensity differences in emotional stimuli remain unclear. To our knowledge, no studies to data have investigated the different emotional responses between individuals with high and low self-esteem by manipulating the intensity of emotional stimuli.

The sociometer model put forward by Leary and his colleagues [14,15] holds that self-esteem is a motivational-affective system that enables people to supervise the extent to which they consider themselves to be valued. Low self-esteem is said to partly stem from continuous social exclusion and negative feedback [14,15]. Because individuals with low self-esteem rely heavily on social approval to feel good about the self [16], the goal of feeling valued and discerning whether others truly care for them is likely to be chronically active [17]. In addition, self-doubts and expectations of rejection which reflected in individuals with low self-esteem make rejecting experiences more painful because they pose a greater proportional loss to a more vulnerable sense of worthiness [14,17]. Hence, according to the model of relationship-specific sociometer [17], individuals with low self-esteem are likely to have a prevention oriented cognitive-motivational system that quickly detects rejection, potential relational devaluation, attend to negative social cues, as well as overreact with negative affect [3,4,8,18–20]. As a result, they are more likely to engender extremely strong experiences of negative affect when processing negative cues during their lives. On the contrary, high self-esteem individuals should barely have the need for a defensively calibrated social alarm system and thus may care less about minor threats in daily life [17].

Consistent with the sociometer model, in a laboratory study with normative stress paradigms, people with low self-esteem released more cortisol after experiencing failure and negative comments [21]. Moreover, recent study has shown that low self-esteem is related to biased attention concerning social exclusion. It is found that people with low self-esteem are likely to be on a higher attentional alert for cues that link to exclusion compared with those that relate to acceptance [3,4]. These behavioral findings have been reinforced by ERPs studies. For example, Li and his colleagues [5,6] found that cues of exclusion produced higher P2 and N2pc components response among people with low self-esteem.

On the other hand, we can also conceive in accordance with sociometer theory that the threshold for detecting positive cues is different among people with different degrees of self-esteem. More specially, individuals with low self-esteem may predispose to react more mildly to positive cues and thus they have an attenuated effect on positive mood. On the contrary, high self-esteem people may respond more actively to moderate cues of acceptance. In accordance with this view, previous research has found that low self-esteem individuals have a lower than normal *set point* for their emotions [22,23]. In contrast, high self-esteem individuals have a higher set point and may view positive affect as typical of them. Thus, individuals with high self-esteem typically enjoy higher positive affectivity and try to keep that affect; while individuals with low self-esteem typically have lower positive affectivity and tend to dampen it [23,24]. In addition, research on buffer hypothesis of self-esteem also indicates that the differences between people with different degrees of self-esteem appear under relatively positive conditions instead of under stressful conditions [25,26]. Although these studies imply that people with high versus low self-esteem respond differently to positive events, how self-esteem difference in the threshold of positive emotion elicitation has yet to be directly investigated.

Thus, the present study investigated the different emotional responses between individuals with high and low self-esteem by manipulating the intensity of emotional stimuli. Specially, individuals with low self-esteem may be sensitive to mild negative stimuli, this, however, may not necessarily be true for individuals with high self-esteem. Similarly, individuals with low self-esteem tend to set higher thresholds in terms of responding to positive stimuli than those with high self-esteem. For this purpose, this study adopted a revised visual oddball task and ERPs measures. As emotional responses in daily life are mostly induced by unexpected and accidental factors in non-emotional states, a distractive task irrelative to emotion (i.e., oddball task) is adopted, which requires participants to draw a distinction of non-emotional standard/deviant by pressing two response keys, regardless of the feelings of deviant stimuli. This process makes the obtained emotional responses more similar to the natural environment [11,27]. Furthermore, ERPs technique was used due to excellent (millisecond) temporal resolution. ERPs help unravel how different cognitive processes, indexed by different components, reflect the influence of self-esteem on emotional responses.

Some studies have examined the effects of emotion on several ERPs components by the oddball task such as the early frontal-central components of P2 [28,29] and N2 [11,30,31], and the late component of parietal P3 [32]. The early P2 and N2 components demonstrate early perceptual and attention patterns. For instance, the P2 amplitude, recorded at frontal and central sites, is higher in terms of reactions to unpleasant pictures compared with pleasant ones beginning around 200 ms post-stimulus. Moreover, a centrally peaking N2 represented the change of attention toward possibly vital stimuli in the oddball tasks [29,30]. Furthermore, brain processing bias for deviant stimuli may as well emerge later on, which requires conscious and controlled processes [31]. This is clearly evidenced by the enhanced P3 response to deviant stimuli in ERPs studies using the oddball task [30,32].

Therefore, this study looked into the effects of self-esteem on the positive and negative sensitivity of the brain to stimuli of different intensity through an oddball task and EPRs measures. In light of the sociometer theory, it could be hypothesized that, compared with people with low self-esteem, those with high self-esteem would be particularly sensitive to changes in valence intensity of positive stimuli yet less sensitive to negative stimuli of diverse strengths. In more detail, it was possible to observe that the P2, N2 and P3 amplitudes, associated with attentional orientation and controlled cognitive processing, were more pronounced in pleasant conditions for high self-esteem people in comparison with low self-esteem people. However, if people with high self-esteem were truly less likely to be affected by unpleasant events than low self-esteem people, they would exhibit less ERPs differentiation for negative stimuli of different strengths.

## Materials and methods

### Participants

Selection of the participants was made out of a collection of 220 college students on the bases of scores obtained by them on the Rosenberg Self-Esteem Scale [33]. The Rosenberg self-esteem scale constitutes 10 items, for instance “On the whole, I am satisfied with myself” that gets rates on a 4-point range starting from 1 suggesting strong disagreement, to 4 that suggests strong agreement. It makes assessment of a person’s general appraisal of one’s self-worth. The Cronbach’s α in the current research is .91. On the bases of the points obtained by them, persons grabbing the scores in the upper 20th percentile of the distribution were classified being the group having a high self-esteem. On the other hand, ones scoring in the lower 20th percentile of the distribution were kept in the category of the group having the low self-esteem. Out of these categories, 27 participants with low self-esteem (11 males; 18–23 years, mean age = 20.33, *SD* = 1.21) and 27 participants with high self-esteem (13 males; 18–24 years, mean age = 20.81, *SD* = 1.24) were awarded invitation by the voluntary principal for attending the electrophysiological study. We based the number of participants required for the experiment on previous studies that investigated self-esteem difference by measuring ERP response [5,34,35]. Furthermore, because small sample size undermines the reliability of results, scholars now recommend a large sample size in neuroscience studies [36]. Thus, a relatively large sample size (n = 54) was used in this study.

The mean score for the low self-esteem group was 20.85 (SD = 2.13), and that for the high self-esteem group was 33.04 (SD = 2.35). The difference in the participant gender composition of the group is not significant (*χ*^*2*^ [1] = .30, *p* > .58). Moreover, the addition of participant gender in the analyses didn’t significantly change the findings hereunder. Therefore, participant gender will not be discussed further. Every participant in the groups was right-handed, having either normal or corrected vision, together with having no neurological disease. They received payment for their participation as well. The research was approved by the Chengdu Medical College Institutional Review Board and written informed consent was obtained from all participants. All experiments were performed according to relevant guidelines and regulations.

### Materials and task

The current research involved two oddball experimental sessions (positive and negative sessions) whereby each of them comprised 4 blocks consisting of 100 trials, wherein, every block included seventy standard as well as thirty deviant (categorized into three conditions) images. The images were taken from the Facial Expression of Emotion: Stimuli and Tests [37] series set where faces from the Ekman [38] series have been morphed from a neutral expression to fully emotive in 10% intervals. Black and white pictures (15 × 10 cm) of positive (happy) and negative (anger) expressions were used from the same actress and were presented separately using an oddball sequence with neutral faces as the standard and intensities of 40%, 70%, and 100% as targets (10% probability each).

**Fig 1:**
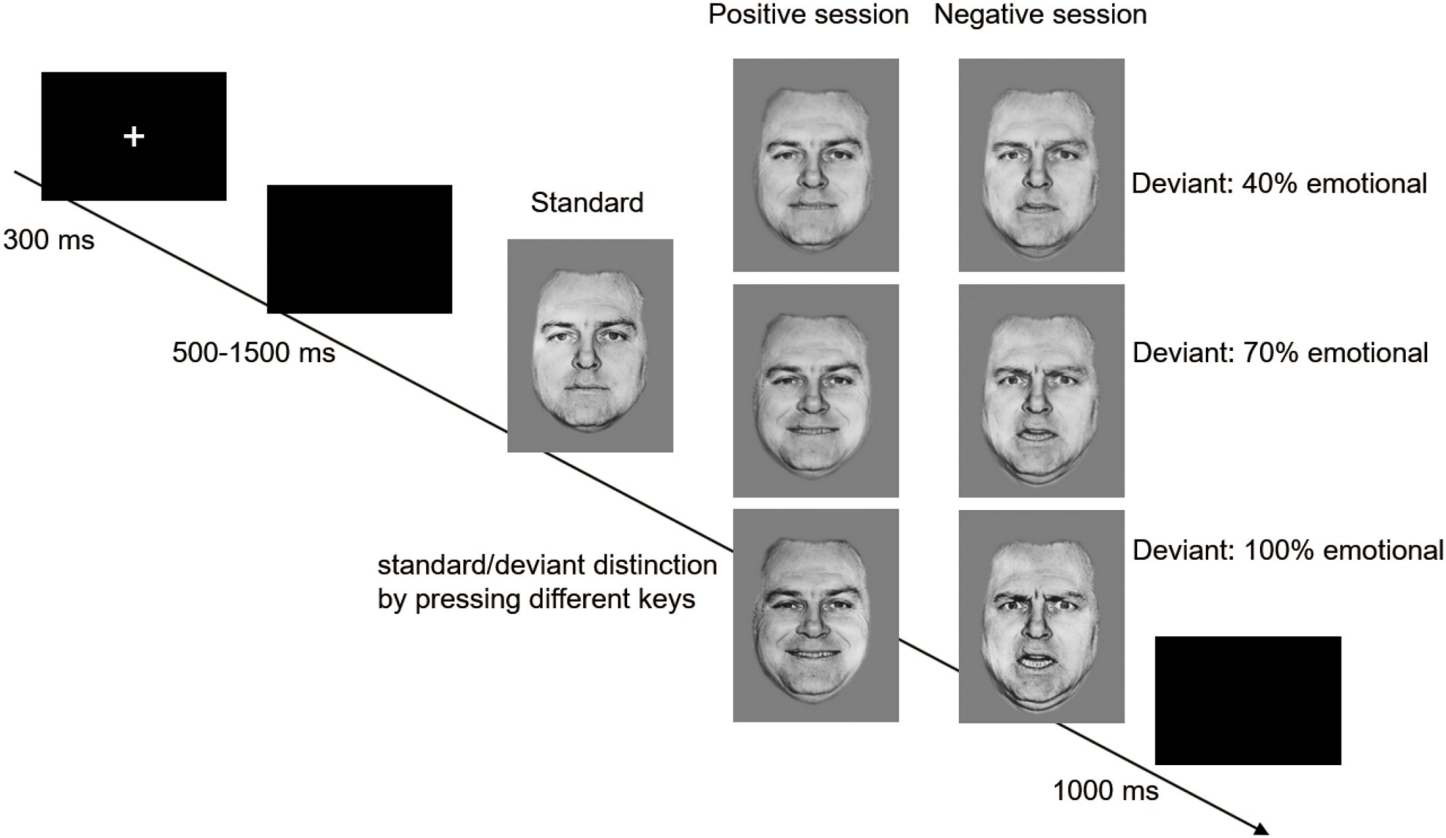
Schematic illustration of the experimental procedure and the stimuli examples. Each trial presented a single stimulus. In a session, a standard stimulus (neutral face) was presented in 70% of the trials, while stimuli (happy or anger faces of diverse intensities) in each deviant condition were presented in 10% of the trials.

Subjects were asked to seat facing a monitor, at a distance of approximately 150 cm from the screen, having the horizontal and vertical visual angles being less than 6^◦^. For the purpose of avoiding fatigue, 2 minutes breaks were given to the participants subsequent to every block. Moreover, they were also provided with the accuracy of their responses as a feedback to gauge their performances. E-Prime software (Psychology Software Tools) presented the stimuli and recorded the responses. Start of every trial was taken through a 300 ms presentation of a tiny white cross. Thereafter, a blank display with randomly varying duration between 500 and 1500 ms was in the following by the onset of face stimulus. Instructions were provided to participants for pressing the ‘‘F” key on the keyboard by using the left index finger as quickly and accurately as possible in a case the standard images appeared. On the other hand, they were asked for pressing the ‘‘J” key by using the right index finger in the case of appearance of the deviant image. Termination of the stimulus image took place either through the key press or on the elapse of a time period of 1000 ms. Every responses were followed by 1000 ms of a blank screen (see Fig 1 for the experimental procedure). The sequence of standard and deviant images was randomized. The order of positive and negative sessions was counterbalanced across participants.

### Electrophysiological recording and analysis

Brain electrical activity was recorded from 32 scalp sites using tin electrodes mounted in an elastic cap (Brain Products, Germany), with the average references on the left and right mastoids for offline ERP computation [39] and a ground electrode on the medial frontal aspect. The vertical and horizontal electrooculogram (EOG) were recorded as electrodes placed above and below and from left versus right orbital rim respectively. The EEG was filtered between 0.01 and 30 Hz. The impedance of all electrodes was less than 5 kΩ. EEG and EOG were amplified with a DC ~ 100 Hz bandpass and digitized at 500 Hz/channel for offline analysis. EOG artifacts were corrected based on Independent Components Analysis. After ocular correction, we conducted filters, segmentation, and baseline correction. After that, any trials containing artifacts (± 80 μV or greater) were automatically rejected.

**Fig 2:**
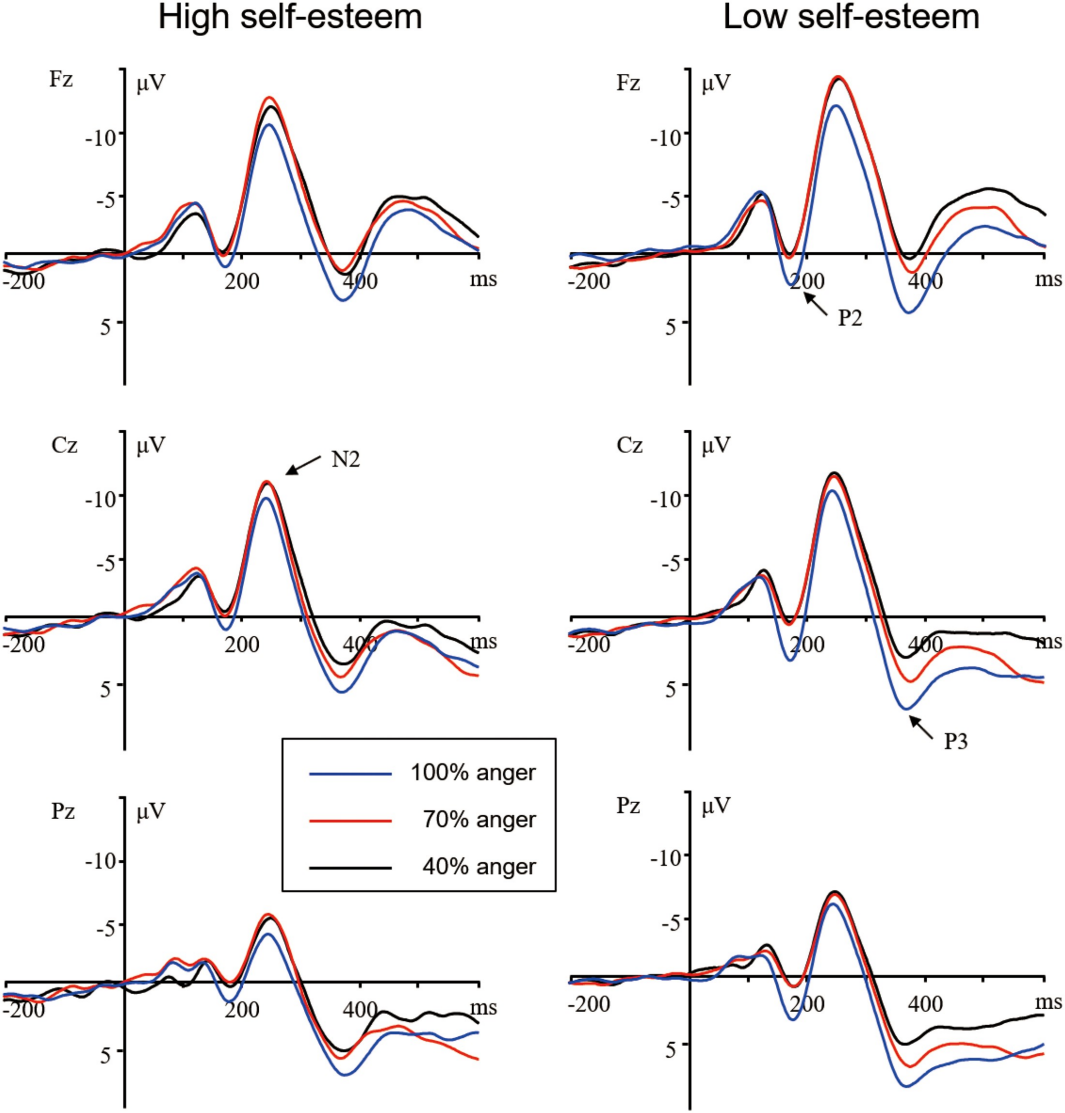
Averaged ERPs for high self-esteem (left) and low self-esteem (right) participants during the 100%, 70%, and 40% face conditions in the negative session.

**Fig 3:**
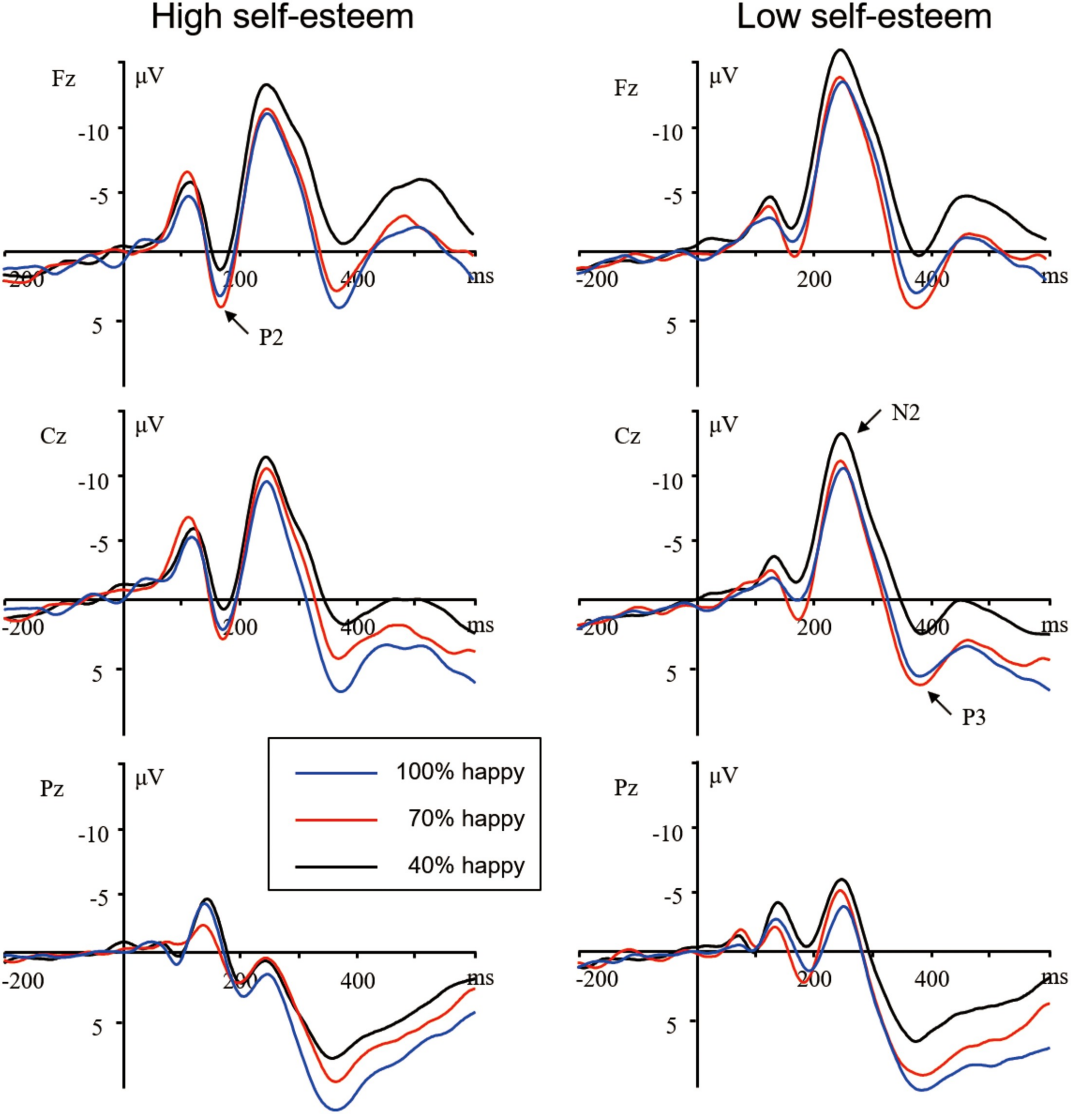
Averaged ERPs for high self-esteem (left) and low self-esteem (right) participants during the 100%, 70%, and 40% face conditions in the positive session.

EEG activity for the correct response in each condition was averaged separately. ERP waveforms were time-locked to the onset of stimuli and the averaging epoch was 1000 ms, including a 200 ms pre-stimulus baseline. As is shown by the ERP’s grand average waveforms (see Figs 2 and 3), each emotion condition, irrespective of levels of self-esteem, elicited apparent frontal and central P2 and N2 components, and broadly distributed P3 activity. This is consistent with the scalp distributions reported by previous ERP studies using affective pictures [11,28]. Thus, the amplitudes (baseline to peak) and peak latencies of the P2 (140–200 ms), N2 (220–300 ms), and P3 (300–500 ms) were measured and analyzed. We selected the following six electrode sites for statistical analysis of P2 and N2 components: F3, Fz, F4 (three frontal sites), C3, Cz, and C4 (three central sites). A repeated measures ANOVA of the amplitudes and latencies of these components was conducted with the following repeated factors: valence (happy, anger), intensity (40%, 70%, 100%), caudality (frontal and central), and laterality (left, midline, and right). Self-esteem was used as a between-subjects factor. In addition, because P3 activity was distributed broadly across both anterior and posterior regions, the analysis of the P3 component also included the three parietal sites (P3, Pz, and P4), along with the six sites above. Since the present study focused on the effect of self-esteem on brain susceptibility to positive and negative stimuli of diverse emotional intensities, we focused the statistical analysis on the two-way interaction between stimulus intensity and valence. For brevity reasons, only effects involving the valence and intensity are described in this report. Statistics were adjusted by the Greenhouse–Geisser method for non-sphericity if the number of factor levels exceeded two. In the present study, uncorrected degrees of freedom but corrected *p* values are reported.

## Results

### Behavioral data

The repeated measures ANOVA of the reaction times (RTs) data (see Table 1), with valence and intensity as repeated factors and self-esteem as a between-subject factor, yield a significant main effect of intensity, *F*(2, 104) = 12.15, *p* < .001, η^2^_p_ = .19. Both 70% (570 ± 12 ms, *p* < .001) and 100% (573 ± 13 ms, *p* = .003) faces elicited shorter RTs than 40% faces (594 ± 13 ms), while there was no difference between 70% and 100% conditions (*p* > .46). The ANOVA of the accuracy rate showed a significant main effect of intensity, *F*(2, 104) = 111.06, *p* < .001, η^2^_p_ = .68. The main effect of intensity resulted from higher percentage of correct responses for 70% (96.2%) and 100% (97.7%) faces as compared to 40% (81.4%) faces. No other main or interaction effects were significant for RTs and accuracy.

**Table 1:** Descriptive statistics for response times and standard deviation for each condition in the positive and negative sessions.

	High self-esteem	Low self-esteem
Positive session (ms)		
40%	594.59 (121.77)	583.36 (95.06)
70%	571.27 (98.95)	567.94 (94.74)
100%	557.92 (78.80)	573.67 (115.55)
Negative session (ms)		
40%	594.39 (107.83)	603.07 (82.98)
70%	567.34 (88.87)	574.91 (85.94)
100%	576.43 (101.45)	585.51 (107.80)

### ERP data

***P2***. The ANOVA on P2 amplitudes showed a significant main effect of intensity, *F*(2, 104) = 26.73, *p* < .001, η^2^_p_ = .34, and a significant interaction effect between valence and intensity, *F*(2, 104) = 18.24, *p* < .001, η^2^_p_ = .26. The simple-effect analyses of the two-way interaction showed a significant intensity effect in anger faces, *F*(2, 106) = 21.82, *p* < .001, η^2^_p_ = .29, with larger amplitudes recorded for 100% faces (2.43 ± .54 μV) than for 70% (.67 ± .50 μV) and 40% (.65 ± .51 μV) faces. Additionally, the intensity effect was also significant in happy faces, *F*(2, 106) = 24.31, *p* < .001, η^2^_p_ = .31, with larger amplitudes recorded for 100% (2.25 ± .69 μV) and 70% faces (2.94 ± .74 μV) than for 40% faces (.39 ± 58 μV). There was no difference between 70% and 100% conditions (*p* > .07). No other main or interaction effects were significant for P2 amplitudes, and there were no significant effects for P2 latencies, either.

***N2***. The ANOVA of N2 amplitudes showed a significant main effect of intensity, *F*(2, 104) = 13.86, *p* < .001, η^2^_p_ = .21. More importantly, there was a significant self-esteem × intensity × valence × laterality interaction, *F*(4, 208) = 5.70, *p* = .003, η^2^_p_ = .10. Simple-effects ANOVAs dissecting this interaction produced a self-esteem × intensity × valence interaction for midline sites, *F*(2, 104) = 5.61, *p* = .007, η^2^_p_ = .10. This interaction was only significant for anger faces, self-esteem × intensity, *F*(2, 104) = 5.04, *p* = .01, η^2^_p_ = .09. The analysis of interaction between self-esteem and intensity revealed a significant main effect of intensity in low self-esteem subjects, *F*(2, 52) = 4.94, *p* = .01, η^2^_p_ = .16. 100% anger faces (−11.47 ± 1.03 μV) elicited greater positivity than 70% (−12.65 ± 1.17 μV, *p* = .04) and 40% anger faces (−13.47 ± 1.08 μV, *p* = .01), while the amplitude differences between 70% and 40% stimuli were not significantly different (*p* > .20). In contrast, the intensity effect was not significant in high self-esteem subjects, *F*(2, 52) = 1.34, *p >* .27 (see Fig 2). In addition, the analysis of N2 latencies showed no significant effects involving intensity or valence.

**Fig 4:**
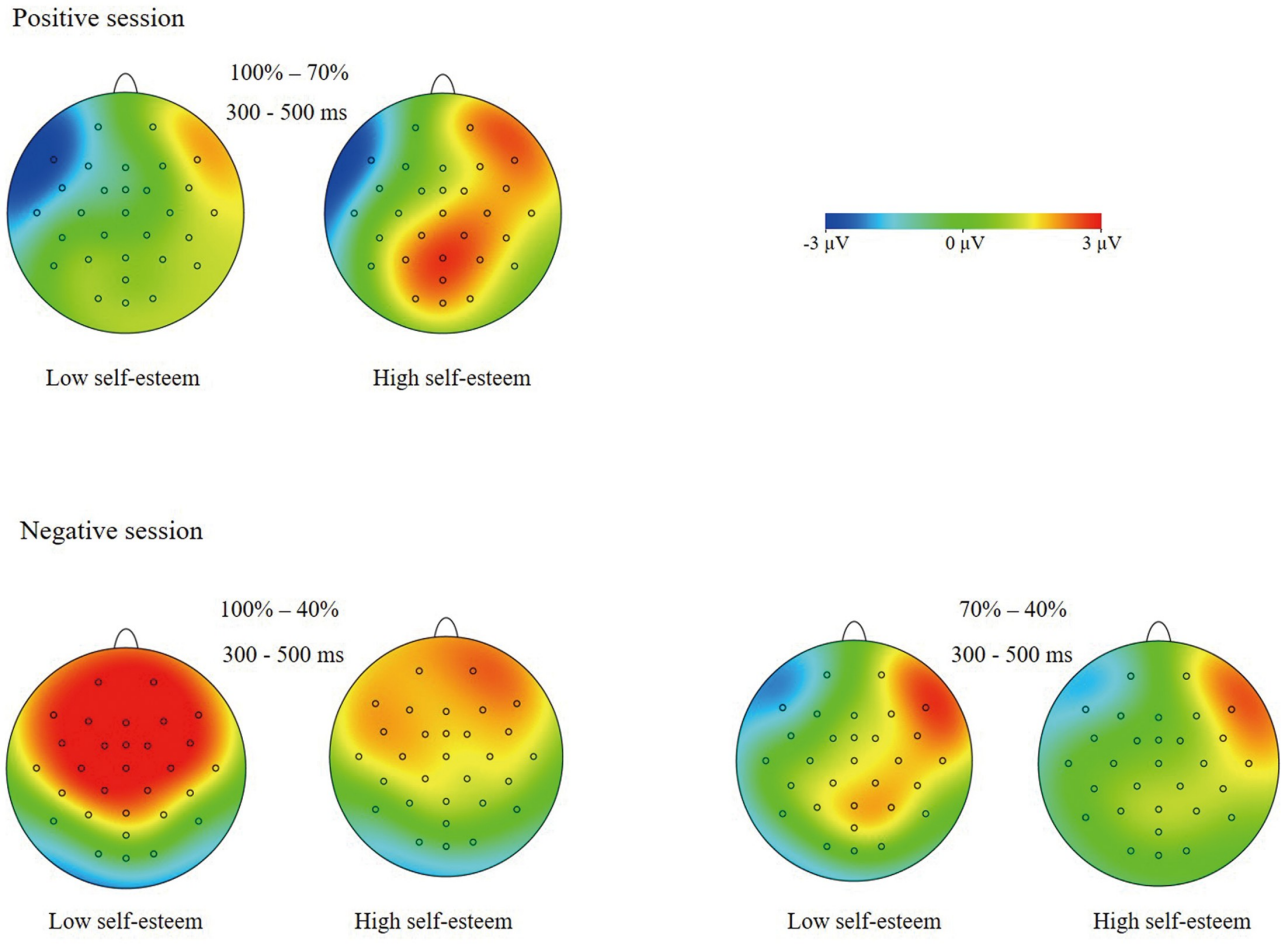
**(Top)** Topographical maps of the amplitudes difference between 100% and 70% happy faces (across 300–500 ms) in high and low self-esteem participants. **(Bottom)** Topographical maps of the amplitudes difference between 100% and 40% anger faces (across 300–500 ms), and between 70% and 40% anger faces (across 300–500 ms) in high and low self-esteem participants.

***P3***. The ANOVA of P3 amplitudes showed a significant main effect of intensity, *F*(2, 104) = 37.64, *p* < .001, η^2^_p_ = .42, and significant self-esteem × intensity interaction, *F*(2, 104) = 3.54, *p* = .03, η^2^_p_ = .06, intensity × valence interaction, *F*(2, 104) = 7.39, *p* = .001, η^2^_p_ = .12, self-esteem × intensity × valence interaction, *F*(2, 104) = 9.45, *p* < .001, η^2^_p_ = .15. We analyzed the simple effects by breaking down the self-esteem × intensity interaction in the happy and anger sessions. The analysis showed a significant interaction of self-esteem and intensity in the happy session, *F*(2, 104) = 4.17, *p* < .02, η^2^_p_ = .07. The simple-effect analyses of the two-way interaction revealed a significant intensity effect in high self-esteem subjects, *F*(2, 52) = 25.03, *p* < .001, η^2^_p_ = .49. 100% happy faces (8.50 ± 1.29 μV) elicited larger amplitudes than 70% (6.98 ± 1.27 μV, *p* = .003), which, in turn, elicited larger amplitudes than 40% happy faces (4.73 ± 1.20 μV, *p* < .001). The simple-effect analysis also revealed an intensity effect in low self-esteem subjects, *F*(2, 52) = 33.95, *p* < .001, η^2^_p_ = .57. Distinct from high self-esteem individuals, low self-esteem subjects did not show significant amplitude differences between the 100% (6.57 ± 1.29 μV) and 70% happy conditions (7.06 ± 1.29 μV, *p* > .24), despite larger amplitudes recorded for 100% (*p* < .001) and 70% (*p* < .001) than for 40% happy faces (3.68 ± 1.18 μV; see Figs 3 and 4).

However, the ANOVA of P3 amplitudes conducted in the anger session also showed a significant self-esteem by intensity interaction, *F*(2, 104) = 7.53, *p* = .001, η^2^_p_ = .13. The simple-effect analyses of the two-way interaction revealed a significant intensity effect in low self-esteem subjects, *F*(2, 52) = 12.53, *p* < .001, η^2^_p_ = .33. 100% anger faces (8.29 ± 1.33 μV) elicited larger amplitudes than 70% (7.26 ± 1.34 μV, *p* < .05), which, in turn, elicited larger amplitudes than 40% anger stimuli (5.16 ± 1.35 μV, *p* = .006). In contrast, the intensity effect was not significant in high self-esteem subjects, *F*<1, *p* > .57 (see Figs 2 and 4). Additionally, the analysis of P3 latencies showed no significant effects involving intensity or valence.

## Discussion

This study reveals that self-esteem has a great impact on brain responses to positive and negative stimuli with different emotional intensities. Although both groups showed significant emotional electrophysiological effects for 100% and 70% happy faces in P3 amplitudes, only high self-esteem people showed significant intensity effects for 100% compared to 70% happy stimuli in this component. On the other hand, only people with low self-esteem exhibited pronounced intensity effects for anger faces in P3 amplitudes. Moreover, only people with low self-esteem displayed significant intensity effects for 100% anger faces compared to both 70% and 40% anger stimuli in N2 amplitudes at central sites.

In the time windows before 200 ms, a prominent P2 component came into being. The P2 peaked around 160 ms and its amplitudes were distributed across frontal and central sites, which was in line with the morphology of P2 that related to attention found in previous emotion studies [28]. Frontal P2 is suggested to be connected with stimulus-driven attention [29], and greater attention paid to pronounced stimuli can enhance its amplitudes [28]. Activation of this component indicates rapid detection of significant stimulus characteristics [11,40]. A significant emotion intensity effect was identified for P2 amplitudes, in positive as well as negative sessions. Both groups demonstrated greater amplitudes for 100% and/or 70% emotional stimuli compared with 40% stimuli. This suggested emotionally salient stimuli resulted in improved early visual attention [29,41] and this enhancement was similar for people with either low or high self-esteem. However, we did not observe significant emotion intensity by self-esteem interaction for P2 amplitudes in both positive and negative sessions. Therefore, the influence of self-esteem on emotion response to positive as well as negative stimuli of different strength levels may emerge later. This disagrees with a previous study that demonstrated low self-esteem individuals exhibited higher P2 amplitudes for disgust faces relative to neutral faces compared with those with high self-esteem during an attention shifting task [5]. A key difference between this study and previous researches in this area [5] was that the participants were required to implicitly process the emotion content of the pictures. Previous studies on other personality constructs (e.g., depression, need for power) suggested that early attention was dependent upon explicit encoding of the emotional elements in the materials [27,42,43]. Thus, the non-emotional standard/deviant distinction task might facilitate participants to distract attention from emotion stimuli and lead to reduced brain reactions to these stimuli during early processing stages [44]. Future studies had better test this speculation by taking two attention tasks, one encouraging explicit processing of emotion and one not.

In the time interval of 220–300 ms, there was a prominent N2 component whose amplitudes were biggest through the central and frontal sites, matching the oddball N2 prototype [30,45]. The appearance of N2 activity represents voluntary attention orienting to deviant stimuli, with initial access to conscious processing resources [46]. In this time interval, individuals with low self-esteem showed pronounced emotion intensity effects for anger stimuli at midline sites through frontal and central sites, whereas people with high self-esteem demonstrated no emotion intensity effects in this time interval. This result suggested that people with low self-esteem were more sensitive to negative cues compared with people with high self-esteem, so that only people with low self-esteem directed enhanced voluntary attention to 100% anger stimuli. The present finding was in line with a great deal of earlier studies that showed people with low self-esteem released more cortisol [21] and kept more alert to negative social cues [3–5]. This also agreed with the sociometer theory suggesting that people with low self-esteem tended to show more sensitivity to negative cues and to overreact with negative affect [14]. However, no significant self-esteem by emotion intensity interaction on N2 amplitudes was observed in the positive session, which might be due to the functional importance of oddball N2 in indicating the vigilance to biological significant stimuli [31]. As the deviant stimuli in the positive session presented no threats or other important biological elements, effects of self-esteem on the alerting and orienting response may diminished in comparison with those in the negative session.

In addition, the P3 peaked later than 300 ms post-stimulus onset, and meanwhile its largest peak amplitudes were recorded at parietals regions. The attributes of the P3 observed in this study fitted the function of parietal P3 in reflecting conscious processing relevant to cognitive evaluation about the meaning of stimuli [30,47]. The significant interaction of self-esteem with emotion intensity for P3 amplitudes was observed in the positive session. Among the high self-esteem individuals, 100% condition gave rise to enhanced P3 amplitudes compared with 70% condition, which, in turn, led to larger amplitudes compared with 40% condition. This suggested that individuals with high self-esteem were sensitive to intensity discrepancies in positive stimuli and reacted differently to positive stimuli different in intensity. Conversely, despite enhanced amplitudes in P3 component for 100% and 70% happy stimuli compared with 40% stimuli, individuals with low self-esteem reacted the same way to positive stimuli of 100% and 70% strengths. Therefore, they were insensitive to emotion intensity discrepancies in positive stimuli. In addition, enhanced P3 amplitudes in individuals with high self-esteem for positive stimuli probably indicated enhanced positive emotional induction. As stated above, P3 reflects elaborated cognitive evaluation of stimulus meaning. Cognitive assessment is thought to play an important role in generating emotion and regulating the intensity of emotions [48,49]. Individuals with high self-esteem usually have higher positive affectivity and try hard to keep that affect by emotion regulation strategies, whereas individuals with low self-esteem have lower positive affectivity and try to dampen it [23,24].

On the other hand, while low self-esteem individuals exhibited a remarkable emotional intensity effect toward anger stimuli, individuals with high self-esteem did not. According to the sociometer theory, people with low self-esteem are probably to be especially sensitive to negative cues [14]. As a result, they are more likely to experience especially strong negative affect when processing negative cues. By comparison, people with high self-esteem barely have the need for a defensively calibrated social alarm system and thus will react mildly to threats. Therefore, the above results consistently suggested that the higher positive affect in high self-esteem individuals might result from enhanced attention bias for positive stimuli and decreased sensitivity to negative stimuli compared with low self-esteem individuals.

Several potential limitations of the present study should be noted. First, it would have been helpful to control for other individual differences that are related to self-esteem such as depressive symptoms and neuroticism to determine the unique effect self-esteem has on attentional bias. In a previous study, researchers reported 10 significant correlations between self-esteem and mental health, substance use, and life satisfaction in early adulthood, but only three remained significant after statistically controlling for associated demographics and personality traits [50]. Unfortunately, we did not gather this potentially useful information. Second, only anger faces were used to express negative emotion in this study. Research has shown that the other negative emotion, such as rejection, also can attract more attentional resource of people with low self-esteem compared to high self-esteem participants [3,4]. Therefore, future research should extend our finding to other negative stimuli such as rejection related facial expression (e.g., disgust). Third, an extreme groups design was utilized such that participants were screened to participate in the ERP laboratory session who reported extreme self-esteem scores. This is not an ideal approach because the self-esteem scale provides the researcher with a good quantitative measure of self-esteem level and our strategy overrepresented those with extreme levels of self-esteem at the same time that it failed to account for those with moderate levels of self-esteem. Moreover, extreme groups approach has also the potential to heighten the chances of model misspecification or would result in inflated standardized effect size estimates [51].

In conclusion, by using ERP technique and differencing the intensity of emotional stimuli in a systematic way, the research found that people with high self-esteem were more reactive than those with low self-esteem to highly positive stimuli. Moreover, high self-esteem individuals were not easily affected by negative stimuli relative to low self-esteem individuals. These results are important because the increased sensitivity for positive stimuli and the decreased sensitivity for negative stimuli may provide at least a partial explanation for an even closer connection between high self-esteem and positive affect.

## Supporting information

S1 Appendix Behavioral data. (XLSX)

S2 Appendix N2 component of the ERP data. (XLSX)

S3 Appendix P2 component of the ERP data. (XLSX)

S4 Appendix P3 component of the ERP data. (XLSX)
